# Three-Dimensional Model of Mouse Epidermis for Experimental Studies of Psoriasis

**Published:** 2013

**Authors:** A.G. Soboleva, V.V. Sobolev, S.A. Bruskin, A.V. Mezentsev

**Affiliations:** Federal Non-profit Research Institute of Russian Academy of Sciences, N.I. Vavilov Institute of General Genetics, Moscow 119991, Russia

**Keywords:** acanthosis, cell culturing, psoriasis, cornification, qPCR, three-dimensional modeling

## Abstract

Three-dimensional models of skin and epidermis imitate the structure of real tissues and provide accurate information about certain skin conditions, such as psoriasis. A three-dimensional model of mouse epidermis was generated from the epidermal keratinocytes of newborn mice and treated with cytokines. The aim of this study was to evaluate this model as an experimental model of psoriasis and to assess the changes occurring in its structure and gene expression after the exposure to proinflammatory cytokines. Treatment of the three-dimensional model with either interleukin 17 or a combination of tumor necrosis factor and interferon γ was shown to produce morphological changes, which were similar to acanthosis in psoriatic skin. The observed changes in gene expression of metalloproteinases and certain psoriasis biomarkers, such as *mki67*, *krt16 *and *fosl1*, were similar to the changes in patients’ skin. Notably, changes caused by interleukin 17 were less evident than those caused by the combination of interferon γ and tumor necrosis factor. On the contrary, HaCaT cells exhibited no significant changes in the expression of *fosl1 *and had decreased levels of *mki67 *after being treated with a combination of TNF and IFNG. Moreover, treatment with IL17 had no significant effect on *krt16 *and mki67 expression and even reduced the *fosl1 *levels. The findings suggest that artificially generated three-dimensional models of murine skin can be used to study psoriasis.

## INTRODUCTION

Recently, three-dimensional models of skin and epidermis have begun to be used very frequently to test cosmetic products, as well as to treat chronic wounds and burns [1-3]. The use of these models complies with the existing European regulations that encourage researchers to minimize their animal experimentations and demand proof of the innocuousness and effectiveness of their experimental procedures [4, 5]. With respect to other existing experimental models, three-dimensional models of skin and epidermis are of great interest in simultaneously testing the effectiveness of multiple compounds, taking into consideration the metabolical changes that occur during the terminal differentiation of keratinocytes. Moreover, these models can be used to study tissue remodeling in pathological conditions.

Compared to the conventional monolayered cell cultures where cells as presumed are the same, the existing organoleptic tissue models mimic both the intercellular contacts and interactions of cells with the extracellular matrix. They also reproduce the changes in gene expression that occur in real tissues and organs. Thus, 3D models of skin and epidermis can potentially provide more accurate information regarding certain pathological conditions, such as psoriasis. This makes them an irreplaceable tool for experimental studies. Taking into account the fact that many basic researchers have limited access to donor skin, we would like to develop a new experimental approach to assess changes in the proliferation and differentiation of skin cells in psoriatic epidermis. This new approach does not involve human cells.

It is believed that psoriatic skin lesions are developed due to the action of the immune cells that infiltrate a patient’s skin. These cells secrete certain proinflammatory cytokines, such as TN F, IFNG, and IL17 [6]. In turn, the secreted cytokines activate the epidermal keratinocytes, thus causing sufficient changes in their terminal differentiation program and accelerating their proliferation. Taking into consideration this point, we treated an original three-dimensional model of mouse epidermis with either IL17 or a combination of INFG and TN F to detect the changes in the model structure and gene expression that occurred thereafter. Thus, the aim of this study was to verify whether the model was able to exhibit certain morphological changes similar to those occurring in psoriatic plaques and to assess its potential for further experimental studies of psoriasis.

## EXPERIMENTAL

**Collection of skin biopsies**

Samples were taken under local anesthesia using a 4 mm dermatological puncher. Patients selected for this study did not receive any systemic or PUVA/UV therapy for at least one month prior the study. Full skin biopsies from the patients diagnosed with common psoriasis,* psoriasis vulgaris*, were obtained from psoriatic plaques as well as from their uninvolved skin at least 3–4 cm away from any skin lesion. The samples designated for RN A purification were quickly frozen in liquid nitrogen and transported to the laboratory. Weighted skin samples were homogenized using a mortar and pestle avoiding their thawing. The samples were then subjected to RN A purification and qPCR . Samples designated for histology analysis were fixed in formalin. This protocol was approved to study human subjects by the ethics committee at the N.I. Vavilov Institute of General Genetics and complies with the principles of the Declaration of Helsinki and National regulations on research to study human subjects.

**Preparation of primary keratinocytes from newborn mice**

Corpses of newborn mice designated for the experiment were decontaminated in betadine and consequently washed with 70% ethanol, gentamycin, and an isotonic phosphate buffered saline (PBS). Each skin was then peeled, flattened on the bottom of a Petri dish, and incubated with 0.25% trypsin (PanEco, Russia) overnight. Next day, the epidermis was mechanically separated from the dermis and cut with scissors into small pieces. The cut tissue was incubated in KSFM medium (Life Technologies, USA) under moderate stirring for 30 min at 37°C. The cell suspension was then filtered through 70 μm cell strainers (Sigma- Aldrich, USA) and centrifuged at 250 g for 5 min. The precipitated cells were counted and plated at a density of 1.1x10^4^ cells/cm2 into 25cm^2^ flasks covered with collagen (Sigma-Aldrich, USA). The cells were cultured in KSFM medium supplemented with S7 supplement (Life Technologies, USA). The calcium concentration in the medium was maintained at 60 μM to prevent cells from entering the terminal differentiation program. The next morning after plating, unattached cells were removed. This protocol was approved for working with laboratory animals by the Ethics Committee of the N.I. Vavilov Institute of General Genetics. It also complies with the national regulations on research involving laboratory animals.

**Cell culturing**

HaCaT cells were cultured in DMEM supplemented with *L*-glutamin, 10% FBS (PanEco, Russia), and 5% antibiotic-antimycotic solution (Life Technologies, USA).

**Preparation of murine skin equivalents**

Mouse three-dimensional skin models were generated from acellular dermis and primary mouse keratinocytes. Deepidermized dermis was prepared by thermoinactivation of skin samples in PBS (56°C; 10 min). After thermoinactivation, the epidermis was peeled off the dermis [7]. Prior to the experiment, glass rings were installed on the deepidermized dermis. Rings were pressed firmly to form isolated compartments for culturing the cells. Cells were then seeded (3x10 5 cells/cm2) and cultured for three days in a freshly prepared medium. The following medium was used to culture the cells: DMEM and F12 that were mixed at a 3:1 ratio, 5% FBS, 1% antibiotic-antimycotic, 4 mM L-glutamin (PanEco, Russia), adenine (25 μg/mL), ascorbic acid (50 μg/mL), triiodothyronine (1 μg/mL), 1 μM hydrocortisone (Sigma-Aldrich, USA), and 0.2 μM recombinant mouse insulin and 10 ng/mL epidermal growth factor (R&D Systems, USA). EGF (R&D Systems, USA) was added to the medium after 24 h. The medium was changed every other day. On the third day, the rings were removed and the samples were submerged in the medium. On the sixth day, culturing was continued at the air-liquid interface to ensure that the upper surface of each sample was in contact with air. For this step of culturing, the medium was enriched with *L*-serine (1 mg/mL), *L*-carnitine (2 μg/mL), 7 μM arachidonic acid, and 15 μM linoleic acid (Sigma-Aldrich, USA). Moreover, vitamin E (Sigma-Aldrich, USA) was added prior to changing the medium to the final concentration of 0.5 μg/mL. The following proinflammatory cytokines (R&D Systems, USA) were used: TN F (25 ng/mL), IFNG (25 ng/mL), and IL17A (50 ng/mL). The cytokines were added to the medium every other day for four days starting from day 10.

**Purification and analysis of total RNA**

RN A was purified using the TR IZOL method as described earlier [8]. Samples were repurified using the RN easy kit (Qiagen, Germany) if the absorption ratio, A_260_/A_280_, in at least one TR IZOL purified sample was lower than 2.0. The integrity of the purified RN A was assessed electrophoretically in 1.5% agarose gel under non-denaturing conditions.

**qPCR**

The obtained RN A samples were converted to cDNA using the MMLV RT kit (Evrogen, Russia). These samples were subjected to qPCR with predesigned commercial gene expression assays (Life technologies, USA) on an Eco PCR life cycler (Illumina, USA). The results were analyzed with the Eco software supplied by the manufacturer.

**Histology**

For histology analysis, the samples were processed into paraffin blocks. Hematoxylin- and eosin- (H + E) stained sections were assessed to evaluate the histopathological changes.

**Statistical analysis**

Data were represented as means ± SE. The statistical differences between the means were assessed by a one-way analysis of variances and Student’s t test. If* p*-values were less than 0.05, means were considered to be significantly different.

## RESULTS

**Fig. 1:**
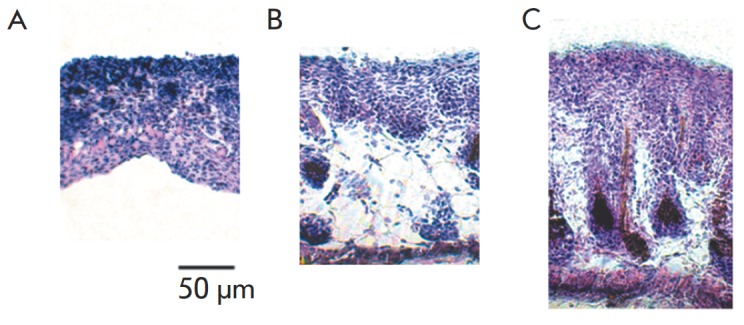
The histological analysis of the most representative TMME samples after treatment with proinflammatory cytokines**.** TMME samples were cultured for two weeks and treated with proinflammatory cytokines starting from the 10th day of culturing: *A *– untreated control; *B *– sample treated with a combination of TNF and IFNG; *C *– sample treated with IL17 (n=6). Harvested samples were embedded in paraffin and stained with hematoxylin and eosin. See the Experimental section for the details

**Fig. 2:**
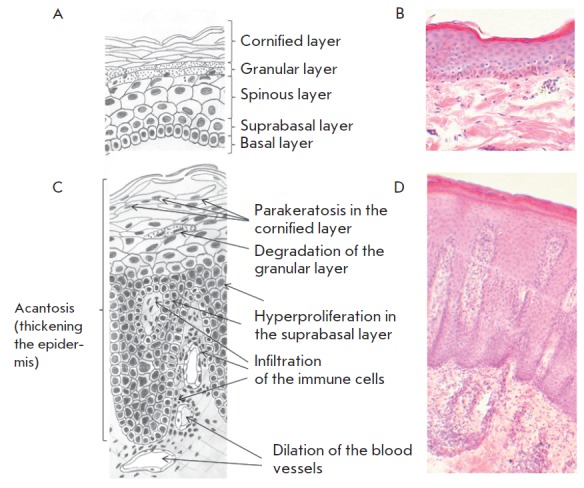
Schematic representation and histological analysis of the psoriatic epidermis**. **The collected tissue samples were embedded in paraffin and stained with hematoxylin and eosin as described in the Experimental section: *A *– schematic representation of uninvolved psoriatic epidermis; *B *– uninvolved psoriatic epidermis stained with eosin and hematoxylin;* C *– schematic representation of a psoriatic skin lesion; *D *– psoriatic skin lesion stained with eosin and hematoxylin

The resulting three-dimensional model of murine skin, TMME (*Fig. 1A*), exhibited a weakly differentiated structure. Unlike healthy human epidermis, TMME was missing the granular layer. Moreover, the transition from the basal layer to the suprabasal layer was not clearly seen. Cornification was also weak, and a suggestion of the corny layer included 2–3 top rows of cells. In uninvolved psoriatic epidermis (Fig. 2A), the basal layer of cells was directly attached to the basal membrane. This cell layer should be the only cell layer where cells are able to proliferate. Starting from the suprabasal layer, keratinocytes gradually changed their shape as they entered the terminal differentiation program. In uninvolved skin, the granular layer separated the cornified anuclear cells and the living nucleated cells of the suprabasal layer (Fig. 2B). In turn, lesional psoriatic epidermis (Fig. 2C) was thickened because of cell proliferation in the suprabasal layer. The evident structural changes attested to certain alterations in the terminal differentiation of cells, such as a delay in the formation of cytoplasmic keratohyalin granules, the degradation of cell nuclei and desmosomes, as well as synthesis of certain biomarkers. Notably, our experimental model exhibited more structural similarities with psoriatic epidermis than normal skin even prior the treatment with proinflammatory cytokines.

**Fig. 3:**
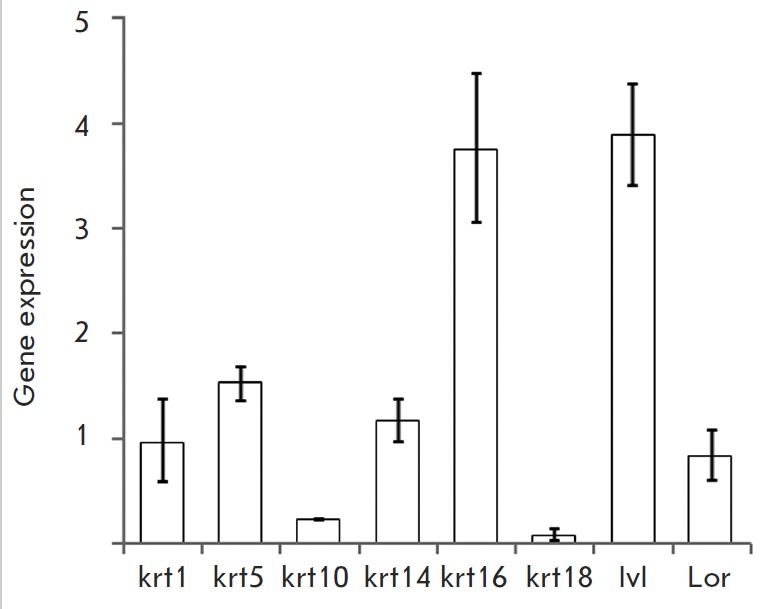
Gene expression of selected cytokeratins and markers of terminal differentiation of the epidermal keratinocytes. The gene expression was assessed by qPCR as described in the Experimental section. Gene expression in TMME was compared to gene expression in the skin of newborn mice (n=3). In untreated control, gene expression levels were considered to be equal to 1

In TMME, the expression of epidermal cytokeratins (*krt1, krt5 *and *krt14*) and the expression of *lor*, which is a marker of the terminal differentiation of epidermal keratinocytes, did not change significantly compared to the skin of newborn mice (Fig. 3). Contrariwise, the expression of *krt10*, *krt18 *and *ivl *was significantly different. The *krt10 *and *krt18 *levels were higher in the skin of newborn mice, while the expression of *ivl *was higher in TMME.

**Fig. 4:**
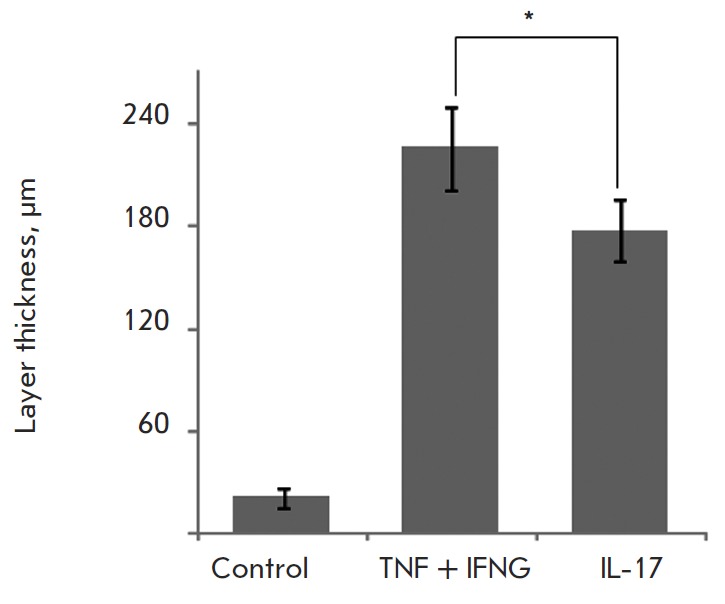
Influence of proinflammatory cytokines on TMME total thicknesses. TMME samples were cultured for two weeks and treated with proinflammatory cytokines starting from the 10^th^ day of culturing as described in the Experimental section

The treatment of TMME with a combination of proinflammatory cytokines TN F and IFNG increased the total thickness of the cell layers composed of living cells by a factor of 1.5 (Fig. 4) compared to the untreated samples. Moreover, the treatment prevented cornification in the top cell layers (*Fig. 1B*) and lowered the cell density in the inner cell layers. Overall, the structure of treated TMME became too fragile compared to the untreated control. The latter complicated the integrity of the generated TMME sections, which were to perform the histological analysis. Notably, the treatment with IL17 produced fewer changes in the total thickness than the treatment with a combination of TN F and IFNG (*Fig. 1C* and Fig. 4). Meanwhile, the total thickness of the model treated with IL17 significantly exceeded the same parameters in the control samples. Thus, TMME exhibited sensitivity to proinflammatory cytokines, such as TN F, IFNG, and IL17 in the same way as the psoriatic epidermis; treatment with a combination of TN F and IFNG produced a stronger response.

**Fig. 5:**
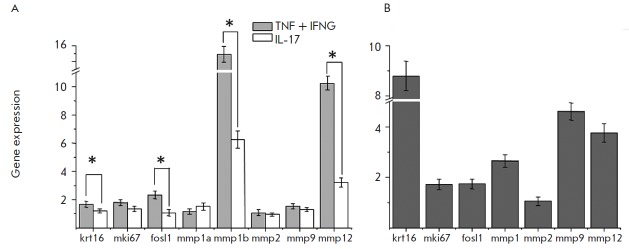
Gene expression observed in TMME treated with proinflammatory cytokines (A) and patients’ skin (*B*). TMME was treated with either a combination of TNF and IFNG or IL17. All samples were subjected to RNA purification, reverse transcription and qPCR analysis as described in the Experimental section. Gene expression in the treated TMME samples was compared to that in the untreated control (n=3)

At the transcriptional level, TMME also exhibited significant changes. These changes were similar to those observed when comparing the lesional and uninvolved psoriatic skin. The levels of certain biomarkers, such as *krt16 *and *fosl1 *as well as the hyperproliferation marker *mki67 *were elevated after the treatment with TN F and IFNG (Fig. 5A). Moreover, TMME also reproduced the expression pattern of metalloproteinases, which included four genes: *mmp1*, *mmp2*, *mmp9,* and *mmp12*. In our previous study [9], we showed that this pattern was highly reproducible in lesional skin. Notably, the changes in the expression of *mki67*, *fosl1,* and *krt16 *induced by the treatment of TMME with IL17 were less evident and did not differ significantly from the control (Fig. 5B). Thus, the combined treatment with TN F and IFNG could be a more significant contributor to the activation of keratinocytes in both psoriasis and TMME.

**Fig. 6:**
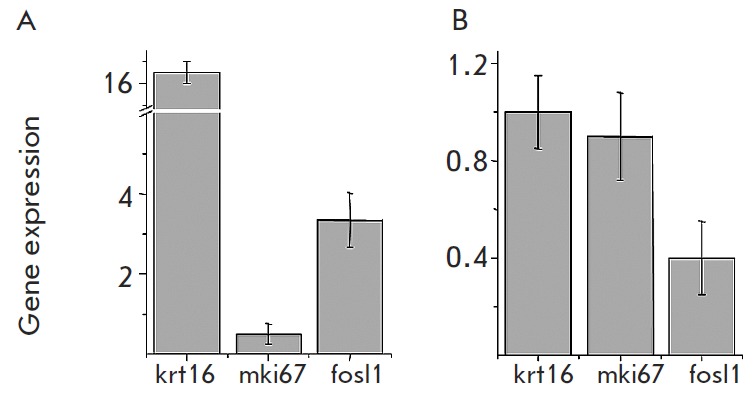
Influence of proinflammatory cytokines on gene expression in HaCaT cells. HaCaT cells were grown to 70% confluence and treated with either a combination of TNF and IFNG (A) or IL17 (B). The cells were subjected to RNA purification, reverse transcription and qPCR analysis as described in the Experimental section. Gene expression in the treated samples was compared to that in the untreated control (n=3)

We have previously demonstrated that treatment of HaCaT cells with proinflammatory cytokines, such as a combination of TN F and IFNG, reproduced the expression patterns of metalloproteinases characteristic of psoriatic lesional skin [10]. The data generated in the present study suggested that TMME treated with TN F and IFNG exhibits similar changes in metalloproteinases expressions (Fig. 5A). Here, we also found that HaCaT cells as a proposed experimental model of psoriasis had sufficient differences with TMME. These differences should be taken into account in further experimental studies involving this type of cells. In particular, *krt16 *was induced in HaCaT cells after treatment with a combination of TN F and IFNG (Fig. 6A). However, these cells failed to induce *krt16* after treatment with IL17 (Fig. 6B). Moreover, neither treatment of HaCaT cells increased *mki67 *and *fosl1* expression as compared to the untreated control (Fig. 6 A and B).

## DISCUSSION

Psoriatic skin lesions emerge through a complex multistage process that involves many signaling mechanisms and requires several kinds of cells. The imitation of pathological skin changes that occur in psoriasis will help to encircle the molecular processes playing an active role in the pathogenesis of this disease. It will also help to clarify the connections between these processes and the clinical symptoms of psoriasis. A number of recent publications have been devoted to the development of new three-dimensional models of human skin and epidermis for different practical needs; their number still continues to grow. The main reason behind the rising scientific interest in three-dimensional models is the ability of an artificial skin to heal severe skin damages. Several three-dimensional models, including the model that uses an endogenous carcass [11] and other models that use natural and biodegradable polymeric materials, such as chitin and chitosan [12], polylactate [13], a combination of amorphous poly (*D*,* L*-lactide) and polyethylene glycol [14], etc. have been developed in Russia. These inventions are helpful in healing burns [11] and venous stasis ulcers [3].

In the most frequently used experimental threedimensional models of skin and epidermis designated for basic research, human epidermal keratinocytes are cultured on a support, such as deepidermized dermis or collagen gel with embedded fibroblasts. In these three-dimensional skin models, either mouse or human cell lines serve as the main source of fibroblasts. Prior to being embedded into the gel, these cells are usually treated with either γ-irradiation or mitomycin C to suppress cell division. In turn, leftovers of cosmetic surgery and circumcision are used as a main source of keratinocytes. Depending on the objectives of a study; three-dimensional skin models may also include macrophages [15], melanocytes [16], and dendritic cells [17]. Thus, the TMME that we are proposing in this study is made of mouse keratinocytes; thus, this model does not rely on any human tissue material or cells.

The use of keratinocytes and deepithelized mouse dermis in three-dimensional models can be beneficial for several reasons. The domestic mouse is the most frequently used laboratory animal. Mice are easy to hold and breed in captivity. Their high fertility and the relatively short gestation period allow one to minimize the external supplies of donor skin. An average litter of mice is typically 6–8 pups and their gestation period lasts only 18.5 days. The use of cells of newborn pups to construct TMME increases the cell yield because of the following rapid decline in the epidermal thickness during the first days of the pups’ terrestrial life.

Several kinds of genetically modified mice, such as transgenic mice [18] and mice with deletions in certain genes [19], can serve as animal models to study psoriasis. In other mouse models of psoriasis, the diseased skin phenotype can be caused by either spontaneous mutations in the genome [20, 21] or by treating wild-type mice with certain chemical agents [22]. The development of these models made some of them very close in details to the clinical features of human psoriasis [18, 22].

Design and development of TMME is in line with European regulations that encourage researchers to minimize their needs in laboratory animals and to develop animal-free models, such as cell cultures and tissue equivalents [4, 5]. In the obtained model, we detected* krt1*, *krt5, *and *krt14*, which contribute to the terminal differentiation of epidermal keratinocytes. Moreover, their expression in TMME and the skin of newborn mice were comparable (Fig. 3). In addition to the cytokeratin genes, we also detected an expression of *ivl *and* lor *in the model, whose expression precedes the cornification process. The elevated *ivl *and *krt16 *expression levels, as well as the lower *krt10 *levels, in TMME suggest that certain changes in the differentiation of epidermal keratinocytes occurred during the culturing. On the contrary, lower *krt18 *levels suggest that unlike the skin of newborn mice, TMME is predominantly composed of a single cell type, and these cells are epidermal keratinocytes.

The results presented in this paper also demonstrate that TMME exhibits certain important similarities with psoriatic plaques. Primarily, TMME was responsive to treatment with proinflammatory cytokines. Treatment with these cytokines thickened the populated cell areas (Fig. 4). Furthermore, this treatment prevented cornification in the upper cellular layers compared to the untreated control (*Fig. 1* A-C). In addition, treatment with these cytokines also caused certain changes in the expression of metalloproteinases,* krt16*, *mki67, *and *fosl1* (Fig. 5A). Notably, the observed changes in the TMME appearance (*Fig. 1* A-C) remind of acanthosis in psoriatic epidermis (Fig. 2B), while the changes in the expression of metalloproteinases,* mki67*, cytokeratin *krt16*, and nuclear protein* fosl1 *in TMME (Fig. 5A), which are specific psoriasis biomarkers, were similar to those observed in psoriatic epidermis (Fig. 5B).

To characterize TMME at the transcriptional level, we selected the genes whose role in the pathogenesis of psoriasis had already been determined. For instance, metalloproteinases MMP1, MMP9, and MMP12 participate in the structural re-arrangement that occurs in psoriatic epidermis [9]. Their activity is crucial for the maintenance of the extracellular matrix and basal membrane, angiogenesis and cell migration within the epidermis, such as the migration of the immune cells, which infiltrate lesional psoriatic epidermis. A shift in the balance between the metalloproteinases in the skin coincides with an aggravation of psoriasis. In turn, an increase in the *mki67 *level is indicative of hyperproliferation of keratinocytes [23]. An elevated *mki67* expression is an important characteristic of lesional psoriatic skin [24]. Cytokeratin *krt16 *is specifically expressed in lesional psoriatic skin (Fig. 5B). Moreover, this cytokeratin is expressed in normal skin at a very low level [25]. *fosl1 *was chosen because its expression correlated with exacerbation of psoriasis and dropped with the beginning of remission [26].

Notably, the treatment with IL17 produced no such evident changes in gene expression compared to the treatment with a combination of TN F and IFNG (Fig. 5A). While the expression of the two preselected genes *mmp1b *and *mmp12 *remained significantly higher compared to the untreated control, the expressions of other genes, such as *krt16*, *mki67*, *fosl1*, *mmp1a*,* mmp2, *and *mmp9*, did not change significantly: i.e., did not exceed 50% of their expression in the control samples.

Unlike humans that have only one MMP1 isoform, mice have two different MMP1 isoforms (MMP1a and MMP1b). The coding genes of *mmp1a *and *mmp1b *localize in the same gene cluster on chromosome 9 [27]. These genes have high sequence homology with each other as well as human *mmp1*. Despite the fact that both genes were discovered more than ten years ago [27], their physiological differences remain unclear. Moreover, both murine *mmp1 *respond similarly to treatment with cytokines and growth factors [28]. The inclusion of both isozymes in our gene selection to verify TMME allowed us to demonstrate that *mmp1a *was capable of producing a stronger response to proinflammatory cytokines. On the contrary, *mmp1b *expression could not exceed the control levels by a factor of 1.6 after either treatment (Fig. 5A).

As compared to TMME, expression of the same genes by HaCaT cells, which have been previously considered to be a conventional two-dimensional cellular model to study psoriasis, has not met our expectations (Fig. 5A and Fig. 6, respectively). For instance, the treatment with TN F and IFNG suppressed *mki67 *expression (Fig. 6A). The different responses of HaCaT and TMME to this treatment can be explained by the deviation from the physiological optimum of HaCaT cells. Notably, the least expected changes in gene expression occurred after the treatment of HaCaT cells with IL17. The expression levels of the two selected genes (*krt16 *and* mki67*) did not change significantly, and the expression of *fosl1 *dropped to a lower level compared to the untreated cells. Thus, a conclusion can be drawn that TMME is more adequate in simulating biomarker expression than a monolayered culture of HaCaT cells.

On the other hand, TMME still has certain differences compared to the other three-dimensional human skin models. In TMME, cell layers are not clearly separated from each other, cornification is insignificant, and cell distribution in middle layers is more diffused than can be expected. Moreover, TMME is missing the granular layer. Unfortunately, we cannot explain these structural ablations yet. However, we believe that they might be caused by fundamental differences in the development of the murine and human epidermis. For instance, the murine epidermis is not completely formed by the time a mouse is born, and certain changes in the content of the TMME cell culture medium will allow us to overcome it.

In order to stabilize the levels of epidermal growth factors we propose to supplement the model with an underlying layer of non-proliferating dermal fibroblasts. To prevent fibroblasts from proliferating, we will treat them with mitomycin C prior to embedding cells into the gel [29]. At early stages of tissue culturing, we will use the inhibitors of GSK3 kinase to slow down the terminal differentiation of epidermal keratinocytes. Previously, one of such agents was used to generate inducible stem cells from the epidermal keratinocytes [30]. At the later stages of culturing, we will add either IL1 or oncostatin M to the culture medium. According to the published data [31, 32], both IL1 and oncostatin M have the ability to induce *S100A *genes. IL1 also stimulates the expression of terminal differentiation markers, such as transglutaminase 1, *Tgm1*, and involucrin,* Ivl*. Thus, this supplementation is expected to improve cornification of the TMME upper cell layers.

## CONCLUSIONS

The findings suggest that artificially generated threedimensional models of mouse epidermis can be used to study psoriasis. These data also demonstrate that activation of mouse keratinocytes is more evident after treatment with a combination of IFNG and TN F rather than IL17.

## Abbreviations

TNF: tumor necrosis factor
IL1: interleukin 1
IL17: interleukin 17
IFNG: interferon γ
TMME: three-dimensional model of mouse epidermis
MMP1: interstitial collagenase (EC 3.4.24.7)
